# A systematic analysis of deep learning in genomics and histopathology for precision oncology

**DOI:** 10.1186/s12920-024-01796-9

**Published:** 2024-02-05

**Authors:** Michaela Unger, Jakob Nikolas Kather

**Affiliations:** 1 Else Kroener Fresenius Center for Digital Health, Technical University Dresden, Dresden, Germany; 2 Department of Medicine I, University Hospital Dresden, Dresden, Germany; 3 Pathology & Data Analytics, Leeds Institute of Medical Research at St James’s, University of Leeds, Leeds, UK; 4 Medical Oncology, National Center for Tumor Diseases (NCT), University Hospital Heidelberg, Heidelberg, Germany

**Keywords:** Deep learning, Histopathology, Genomics, Multimodality, Systematic review

## Abstract

**Background:**

Digitized histopathological tissue slides and genomics profiling data are available for many patients with solid tumors. In the last 5 years, Deep Learning (DL) has been broadly used to extract clinically actionable information and biological knowledge from pathology slides and genomic data in cancer. In addition, a number of recent studies have introduced multimodal DL models designed to simultaneously process both images from pathology slides and genomic data as inputs. By comparing patterns from one data modality with those in another, multimodal DL models are capable of achieving higher performance compared to their unimodal counterparts. However, the application of these methodologies across various tumor entities and clinical scenarios lacks consistency.

**Methods:**

Here, we present a systematic survey of the academic literature from 2010 to November 2023, aiming to quantify the application of DL for pathology, genomics, and the combined use of both data types. After filtering 3048 publications, our search identified 534 relevant articles which then were evaluated by basic (diagnosis, grading, subtyping) and advanced (mutation, drug response and survival prediction) application types, publication year and addressed cancer tissue.

**Results:**

Our analysis reveals a predominant application of DL in pathology compared to genomics. However, there is a notable surge in DL incorporation within both domains. Furthermore, while DL applied to pathology primarily targets the identification of histology-specific patterns in individual tissues, DL in genomics is more commonly used in a pan-cancer context. Multimodal DL, on the contrary, remains a niche topic, evidenced by a limited number of publications, primarily focusing on prognosis predictions.

**Conclusion:**

In summary, our quantitative analysis indicates that DL not only has a well-established role in histopathology but is also being successfully integrated into both genomic and multimodal applications. In addition, there is considerable potential in multimodal DL for harnessing further advanced tasks, such as predicting drug response. Nevertheless, this review also underlines the need for further research to bridge the existing gaps in these fields.

**Supplementary Information:**

The online version contains supplementary material available at 10.1186/s12920-024-01796-9.

## Background

Over the last few decades, precision oncology has emerged as the standard strategy in cancer care. Once a diagnosis is made, precision oncology tailors cancer treatment based on the specific molecular alterations unique to each patient [1]. This is enabled by biomarkers present in the tumor’s morphology or genotype. Biomarkers are biological features that serve as indicators of healthy or pathogenic processes, as well as responses to specific drug treatments [2]. While conventional biomarkers such as histopathological grade or subtype provide preliminary insights into a patient’s disease, there are also more nuanced prognostic and predictive biomarkers available. For example, the presence of lymphocytes in tumor tissue is a useful prognostic biomarker that indicates the course of the disease in various types of cancer [3]. Additionally, predictive biomarkers forecast response to specific treatments. One notable example is homologous repair deficiency (HRD), which can increase patients’ susceptibility for treatment with Poly-ADP-Ribose-Polymerase (PARP) inhibitors [4–6]. Usually, the routine acquisition of patient-specific features is typically limited to a select set of biomarkers, due to high costs, its time-consuming nature, and specialized equipment and expertise required for complex biological assays [7]. Furthermore, many of these advancements remain accessible only to a limited number of cancer patients globally. Consequently, new concepts could help to streamline clinical workflows, enhancing the process from diagnosis to treatment, especially in low- and middle-income countries. Artificial intelligence (AI) tools could play a role in addressing this challenge by offering predictive estimates of biomarkers, thereby supporting clinicians in making informed decisions [8]. Ultimately, in some cases, AI has the potential to bypass the conventional biomarker detection stage entirely [9].

Deep learning (DL), a subclass of AI [10], can extract meaningful patterns from complex data. DL models are neural networks that can undergo supervised training wherein they process input through layers of small units, the neurons. These models generate an output which is then compared to a predefined label. The error which is initially produced during this process is then propagated back through the network, causing updates to the internal parameters, thereby improving the prediction accuracy in the subsequent round [11]. When labels are only partially available or entirely absent, DL models can also be trained using weakly-supervised or unsupervised methods [12, 13]. In oncology, the histopathological phenotype and genetic alterations create an abundance of complex data which can in principle be analyzed with DL. In digital pathology phenotypes from routinely available hematoxylin and eosin (H&E) stained whole slide images (WSIs) serve as a rich data source [14]. In the last years, DL has demonstrated its ability to derive global patterns for cancer diagnosis from WSIs [15, 16], which could offer a more quantitative measure of the disease and enhance diagnostic throughput. Nevertheless, for the majority of cancer types, there remains a need for more comprehensive prognostic and predictive information to refine therapeutic choices. Genomic tests, designed to detect specific alterations in tumor DNA, are part of an arsenal of tests that can yield additional data for clinical decision-making. Traditionally, genomic data has been analyzed using standard bioinformatics pipelines. These are composed of deterministic computer programs, enabling the comparison of alterations in the tumor’s genome either with the patient’s germline genome or a reference genome. In this context, DL holds the potential to replace certain aspects of these traditional pipelines. DL’s ability to discover known patterns in the genomic sequence, but also to identify new ones, could facilitate the accessibility of concealed features within the data. Another recent development is taking place at the intersection of histopathology and genomics – the domain of multimodal models [17]. Technological advancements now enable the simultaneous integration and interpretation of patterns across both data types. Potentially, some patterns in pathology slides might only be meaningful given a known genetic background, or vice versa. As such, multimodal models could offer more comprehensive insights than an independent analysis of either data modality. In conclusion, the application of DL has the potential to advance precision oncology, conceivably making the acquisition of biomarkers quicker and more affordable.

**Fig. 1:**
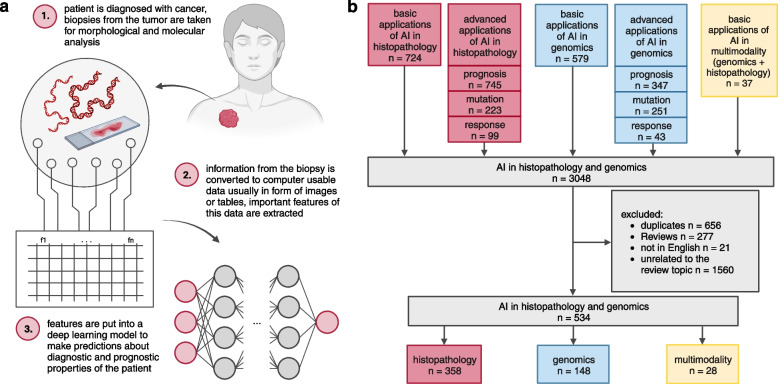
Summary of the motivation and workflow of this review **a**) Workflow of the application of DL in genomics and histopathology. **b**) Flowchart of the systematic search and filter for this review’s literature

Here, we present a systematic review of the literature, covering DL applications in pathology, genomics, and their multimodal combination for precision oncology (Fig. 1a). In order to perform a comprehensive analysis across these expansive fields, we needed to establish a set of criteria related to workflows and biomarker usage in the clinics. Therefore, we divide the literature into six fields of DL application, as established by previous studies [18]. Three “basic” applications: DL for predicting the diagnosis (cancer detection), grading (determining the grade of cancer) or subtype of a tumor; and three “advanced” applications: predicting prognosis (survival probability of the patient), patterns of genetic alterations (such as the detection of driver mutations) or treatment response to a specific therapy scheme or a single medicine [18, 19]. Our systematic analysis resulted in 534 academic publications (Fig. 1b), all of which are enumerated in the Supplementary material and will be explained in the following sections. With this approach, we summarize the integration of DL in these fields, examine overall trends and identify gaps warranting further research.

## Methods

### Article selection criteria

For conducting our systematic review we aimed to adhere to the PRISMA [20] guidelines as closely as possible. However, given that the scope of the review was primarily oriented towards a quantitative analysis of publication numbers, not all screening criteria outlined in the PRISMA guidelines were considered applicable. We designed our query to include publications which employed DL techniques within genomics and histopathology in oncology, and for multimodality in these two fields (Fig. 1b). Additionally, the considered papers had to meet the following criteria: published between the year 2010 and the 16th of November 2023, written in English, and have both title and abstract readily accessible. The considered studies had to utilize DL in at least one of the following six categories: diagnosis, grading, subtyping, prognosis, mutation, and response. In alignment with other publications, we categorized the application areas into basic (diagnosis, grading, subtyping) and advanced (prognosis, mutation, response) tasks. A corresponding flowchart is depicted in Fig. 1b.

### Data extraction

All papers obtained for the PubMed query (queries available in the Supplementary Material) were collected, pooled and annotated, with regards to histopathology, genomics or multimodal data, tissue type, and application class. Rayyan was utilized as a tool for assessing papers and structuring the systematic review process. The applicability of each paper was determined by screening its title and abstract according to our selection criteria. If the relevance of a paper remained ambiguous following this step, we proceeded to a full-text review. Any papers without available full text, or those for which the relevance remained uncertain after full-text review, were discarded. From the publication list we furthermore excluded review articles, duplicated papers, and articles not related to this review topic. Out of scope for this review we defined as papers not related to oncology, not applying DL methods, not applying DL methods in our six categories, utilizing other imaging techniques than bright field microscopy of histological sections, utilizing proteome and metabolome data and/or not using human samples. Certain papers encompassed multiple application classes, which were labeled with all applicable types. A comprehensive list of all selected papers is available in Supplementary Tables 1–3. The search in PubMed displayed some limitations. Specifically, it might not have identified publications that did not include our specified keywords in their title or abstract. Hence, relevant papers that align with our topic of interest may have been omitted. Furthermore, restricting our search to only PubMed as a database could propagate biases to our findings. Nonetheless, in summary, our approach facilitated the discovery of a diverse range of papers, providing valuable insights into the fields of interest.

## Results

**Fig. 2:**
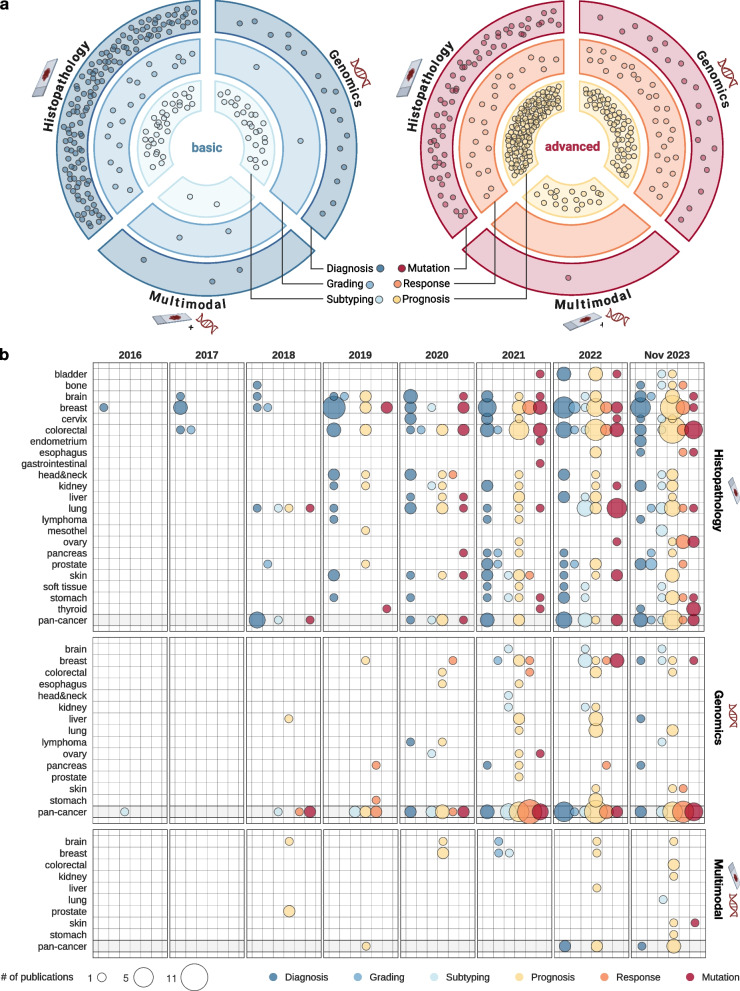
Applications of AI in histopathology and clinical genomics. **a** Number of papers found for every application class in regard to histopathology, clinical genomics and multimodal approaches. Each dot represents one article of the field. The applications were split into basic (Diagnosis, Subtyping, Grading) and advanced (Prognosis, Response, Mutation). **b** Number of papers found for every application class regarding tissue type from 2016 to 2023. The size of the circle is in proportion to the total number of articles while the color displays the application type. Pan-cancer studies are shaded since multiple tissues are combined in them

Initially, we gathered all publications which describe basic and advanced applications from the three key research areas: histopathology, genomics and their multimodal combination. In general, DL is observably more implemented in histopathology than in genomics (Fig. 2a). The task of cancer diagnosis is the primary task tackled by DL-based studies in histopathology, with a total of 128 articles published on this topic. On the other hand, determining tumor grade, a key biomarker, has received less attention in DL studies within histopathology, underlined by 19 publications in this domain. Turning our attention to genomics-based cancer diagnosis, our search yielded 18 papers. Strikingly, only two publications addressed the grading aspect [21, 22]. Regarding tumor subtype, the publication count increased, with 33 for histopathology and 26 for genomics. As anticipated, multimodality demonstrated the fewest publications, with three for diagnosis [23, 24], two for grading [25–27], and two for subtyping [28, 29]. Nevertheless, it is crucial to acknowledge that multimodal models present a significantly higher level of complexity compared to their unimodal counterparts. Given the satisfactory performance of unimodal models in most applications, there has not been a compelling need to utilize more complex multimodal models for simpler tasks.

The landscape shifts when we explore the advanced applications of DL across our research domains (Fig. 2a). Interestingly, the prediction of biomarkers in the form of (driver) mutations is most frequently seen in DL for histopathology, as indicated by the 64 relevant publications. Conversely, genomics accounted for 20 articles in this area. It should be noted that in genomics data, when utilizing whole exome or genome sequencing, driver mutations can be derived without the need of DL, which might account for the lower publication count. Our search revealed only one multimodal publication for mutation biomarkers. Predicting drug response and with this bypassing the traditional biomarker approach, was an area that the histopatho-genomic multimodality did not address at all. However, intriguingly, treatment response became the only DL application for which genomics yielded the highest publication count. Nevertheless, to put this number into perspective, DL in genomics produced 31 publications for this application. This modest publication volume is likely due to the scarcity of data. Publications for drug response in histopathology mostly targeted general therapies like (neoadjuvant) chemotherapy, as opposed to individual drugs. Contrarily, in genomics, cancer cell line screens are the routine datasource for this application type. Moving on to the most prevalent advanced application of DL, prognosis does not focus on biomarker prediction as well, but on directly estimating a patient’s survival probability. Here, histopathology led this area with 125 papers, followed by 60 in genomics. Significantly, 75% of all multimodality models were developed for prognosis prediction. Multimodality thrives in this area as it merges insights from diverse sources, potentially outperforming unimodal models. Yet, the existing number of multimodality publications indicates opportunities for further exploration in this research domain.

Additionally, we investigated publication trends over time and examined the coverage of cancer entities. The first application of DL in histopathology targeted breast cancer diagnosis in 2016 [30], aligning with its status as one of the most prevalent cancers [31]. Furthermore, breast cancer was the focus of the pioneering CAMELYON computational histopathology challenges in 2016 and 2017 [32, 33]. Such challenges could not only emphasize the current trends of the field in terms of state-of-the-art techniques and emerging directions but also highlight knowledge gaps and facilitate collaborations, as well as data and resource sharing. 2017 witnessed further publications in breast cancer, but also in brain and colorectal cancer, alongside with the first grading prediction in histopathology. The field’s substantial growth is particularly noticeable when comparing the publication numbers between 2017 and 2018, approximately displaying a doubling in articles. A seminal paper by Coudray et al. [34], employing DL exclusively to classify lung cancer subtypes and their driver mutations, was published during this period. In 2018, the first four pan-cancer studies in DL for histopathology were published, a novelty considering the substantial data volume required for such research. The first prognosis prediction in this field also occurred in this year. In 2019, ten prognosis-related histopathology DL papers were published, covering a wide range of tissues including breast, colorectal, kidney, and skin, among others. This milestone marked a shift in DL for histopathology, where basic applications no longer dominated the field. 2020 constituted the first year where the number of advanced applications reached the level of basic ones, pushing the field’s knowledge boundaries further. Additionally, this period also witnessed the publication of the first drug response paper in DL for histopathology. Over time similar trends were observable; a surge in prognosis-related publications in 2019 followed the introduction of prognostic applications in 2018. The popularity of mutation prediction, the biomarkers linking genotypic alterations to phenotypic traits in histopathology, was similarly noted in 2020, two years after its introduction. Furthermore, in 2020, pan-cancer publications also expanded their application repertoire. Notably, Fu et al. [21] advanced the field with a publication employing an immense patient cohort for mutation prediction. For the year 2021, we identified a trend for DL in histopathology where specific cancer tissues, including breast, colorectal, skin, and stomach, attracted more research attention across various application types. In contrast, gastrointestinal and ovarian cancers were only explored for the first time, potentially due to prior limitations in cohort size. DL demands large sample sizes for good model performance. As such, more prevalent cancers typically benefit from data availability, while models trained on scarce data are likely to perform poorly. Another factor contributing to the limited number of publications in certain tissue types could be their inherent morphology. Certain cancer entities could form more heterogeneous patterns difficult to recognize for the models, compromising the learning process. Despite these challenges, the growth trend in the field persisted in 2021, yielding 65 publications, only surpassed by the 90 papers published in 2022 and 114 in 2023. When compared to the humble beginnings in the 2010s, this progress is remarkable. Finally, as the last application type, drug response became widely utilized in the field in 2023, not only in single tissue studies such as ovary, colorectal or esophagus but also in pan-cancer studies. This development could open up numerous new clinical applications for DL in computational pathology, ultimately including the use of DL models as companion diagnostics for new drugs. Thus, although the expansion of DL in histopathology might decelerate in the future, automated biomarker prediction from WSIs is anticipated to be translated into clinical workflows in the near future [14].

In DL for genomics, a notable initial observation was the narrower range of investigated cancer entities compared to histopathology. One of the first articles in this field was published already in 2016 [35] focusing on differentiating tumor types from genomic data. Furthermore, early publications targeted pan-cancer studies rather than specific cancer types, likely due to the availability of pan-cancer datasets and the goal of genomics to understand general cancer mechanisms. By 2018, the field had diversified with applications emerging in subtyping, drug response, and mutation prediction, resulting in a total of five publications. Notably, in this year, the first DL model was applied on liver cancers [36]. The publication count grew to nine by 2019, with prognostic applications accounting for 30% of the total. Furthermore, additional cancer tissues, such as breast, and stomach, were investigated. Interestingly, DL for genomics started the exploration of drug response applications earlier, with seven papers published before the debut publication in histopathology-DL in the same field. This is probably the result of greater data generation and time efficiency of cancer cell line studies compared to actual patient data. By 2020, the first diagnostic DL methods appeared in genomics, however, this development did not spark a significant trend in the years to follow. Likewise to pathology, breast cancer remained a dominant research area, with other cancers only occasionally studied. 2021 saw a temporary peak in pan-cancer research for DL in genomics, yielding 24 publications. This suggests that genomic biomarkers may have more sustainability across various cancer types than histopathological ones. In 2022, the interest towards individual prognosis predictions slightly increased. However, the absence of a similar expanding trend in DL for clinical genomics, as observed in histopathology, was a surprising discovery. This suggests that there are hurdles yet to be addressed for the comprehensive integration of DL in this field. Nevertheless, DL could play an immense role in the discovery of novel genetic biomarkers as indicators or targets for individualized therapy in the future.

Multimodal DL research between histopathology and genomics was conducted in the least number of tissue types. This field, only recently established in 2018, saw in its first year the release of three publications. The emergence of studies on brain tumors in this context is likely attributable to changes in the WHO guidelines that now mandate molecular tests alongside pathological sample examinations for patient diagnosis [37]. This requirement probably led DL models to incorporate histopathologic and genomic data to reflect medical workflows. Prostate cancer research also made an appearance in 2018 with two papers both published by Ren et al. [38, 39]. Next to single cancer types, in multimodality, pan-cancer studies are in use as well, as demonstrated by Cheerla and Gevaert [40]. This was the only multimodal DL study published in 2019, suggesting that despite the initial momentum, the field’s overall popularity receded. Perhaps the concept and application of multimodal biomarkers had not been fully developed or realized at that time. Three more articles followed in 2020, two focusing on breast [41, 42] and one on the brain [43], all targeting prognostic biomarkers or direct implications about patient survival. Interestingly, the multimodal research changed paradigms in 2021 and was predominantly directed towards basic applications, namely grading and subtyping. An increase in publications was observed in 2022, including both basic and advanced studies, culminating in a total of seven publications. An expansion was observed in 2023 as well, not only focusing on prognosis predictions but also introducing mutation predictions for the first time in the field. This trend hints at the growing recognition and potential of multimodality in histopathology and genomics, a progress that will probably increase in importance in the forthcoming years.

## Discussion

A first difference between DL in histopathology and genomics emerges when comparing the sheer number of publications. Our literature search yielded more than twice as many articles utilizing DL for histopathology as it did for genomics. Reasons for this behavior are not clear to define but one possible explanation could be data availability. H&E stained slides have been available for decades and are ready to be digitized. Thus, it may be easier to establish DL-appropriate cohort sizes in histopathology than in genomics, given that genomics has only been generating data since the early 2000s. Furthermore, genomics data is not yet routinely collected for every cancer patient which may lead to differences in cohort size as well. Additionally, the origins of DL in histopathology and genomics may play a role in their adoption within these fields. While DL for medical imaging evolved from successful applications in computer vision, its use for sparse, tabular genomic data was less prevalent, facing substantial competition from traditional bioinformatics tools. Another crucial point to consider is the human interpretability of histopathological images compared to genomic information. The human eye can detect distinct patterns in histology, which form the basis for patient diagnosis, making it less abstract and more intuitive than genomic data. As a reflection of this, the field of explainable AI is emerging, aiming to elucidate the black-box properties of DL models. For histopathology, pixel- or region-wise attention maps [44] can be employed to display important areas in the input images. Clinical applications of DL in genomics, on the other hand, are less favored, as the relationships between specific genes and model outputs are often not interpretable. Here, attribution methods like SHAP values [45] can be applied to highlight the most influential features of the data, but this is effective only when the input features are already human comprehensible. Consequently, the application of DL in a medical context may be more straightforward for image-based fields, where practitioners can directly associate the model’s attention with a biological rationale. Furthermore, institutional biases could be stronger for genomic data than for histopathology. Protocols and techniques might lead to more substantial data compatibility issues in genomics than in histopathology. Lastly, legal hurdles could make the distribution of genomic data more challenging than in pathology. Collectively, these factors could contribute to the observed lower utilization of DL in genomics.

Throughout the years, a common theme across all three fields is their expansion in DL. DL in histopathology emerged around the same time as DL in genomics, but in its early years, it primarily focused on basic applications such as diagnosis, grading, and subtyping. This trend could be attributed to the fundamental role that histopathology plays in the medical workflow, forming the basis for these applications. Typically, the initial step in diagnosing cancer patients involves pathology, with genomic data often obtained post-diagnosis, thus rendering its use for genomic-DL in diagnosis redundant in most cases. Additionally, obtaining genomic data from sequencing technologies is more costly than the preparation of H&E tissue slides. This economic factor might contribute to the usage pattern, where genomic data is reserved for challenging cases and advanced applications, while histopathology suffices for diagnosis and basic biomarkers. This trend could explain why genomics predominantly finds application in advanced tasks such as prognosis, prediction of drug response, and mutation prediction. Genomics can potentially offer more profound insights into molecular mechanisms within cancer cells necessary for these advanced tasks. Our observation indicates that drug response was infrequently the objective of DL in histopathology, while it was more extensively covered in genomics. This underlines the possibility that information derived from histopathology might not be sufficient for precise predictions. On the other hand, drug response predictions in genomics were mostly carried out in relation to pharmacogenomics in which cancer cell lines were used. Predicting drug response using fixed cell lines in genomics may be simpler as it lacks the added complexity of real-life scenarios in histopathology, such as the tumor microenvironment and other factors. Regardless, we expect integration of DL in both fields will continue to increase with vast potential for further growth for the future.

A distinct feature of DL in histopathology is the diverse range of cancer tissues studied. In contrast, DL studies in genomics primarily focus on pan-cancer approaches, occasionally focusing on prevalent cancer types such as breast or liver. This could be attributed to cancer type overarching questions posed in genomics, which might necessitate pan-cancer studies. Moreover, genomic data might be less reliant on the specific cancer type and exhibit more consistency. Evidence for this can be seen in the similar molecular alterations, like driver mutations, which are active across different cancer types. This could facilitate the aggregation of different tissue types into larger pan-cancer studies. Nevertheless, genomics could be used in the future to address specific cancer tissues, making predictions more precise and understanding molecular alterations in these cancer types more deeply. Regardless of the rarity of cancer types, patients could still benefit from DL applications. On the other hand, WSIs are highly tissue specific. This means that the absence of pan-cancer studies can be explained by substantially different visual patterns of various cancer types. For instance, breast cancer and brain cancer appear significantly different histologically, as the underlying tissue architecture varies. The patterns recognized by the DL algorithm could become confusing or even contradictory when combined, which may hamper the model’s performance. Consequently, to yield the highest-performing models, DL in histopathology might prefer to keep tissue types separated. However, for future medical applications, it may become necessary to develop models applicable to the broadest possible patient group. Therefore, building DL models that encompass diverse cancer types is arguably more feasible for clinical use than focusing on a single cancer type.

In the realm of multimodality, we observed that the most prominent application was prognosis prediction. Given the complexity of securing a large sample size that includes both genomic and histopathologic data, researchers might have prioritized addressing key challenges such as predicting survival rates or individual patient risks. The articles demonstrated that the combination of histopathology and genomics can embrace synergies between them and make DL predictions more reliable. Moreover, the integration of synergistic data could enable a direct progression to advanced tasks, potentially circumventing the initial biomarker detection stage. Yet, it is unclear how interactions between modalities affect the predictions of these models. In turn, this raises an important question: Is a comprehensive understanding of the model’s inner workings necessary for its clinical deployment, or is exceptional performance justification enough? As this question is open to debate in the scientific community, we still anticipate a serious growth in the coming years for multimodality, since the field’s relative novelty is paired with growing data sources and an immense medical relevance.

In all three areas, there remains a diverse range of unexplored cancer tissues and application combinations. While DL for histopathology needs to enhance its pan-cancer comprehension, DL for genomics must work towards refining its approaches for specific cancer types. For multimodality, we found the most significant gaps concerning advanced applications, with none discovered for drug response and mutation prediction. This presents a substantial opportunity for future researchers to address these vital questions. By understanding how DL models work and elucidating connections between both data types, novel knowledge could be uncovered. This newfound understanding of interplay of the genome and tissue morphology could potentially shift our perception of fundamental biological processes behind cancer development.

## Conclusion

In this review, we have explored applications of DL in histopathology and genomics. Evidently, the rise of DL in these fields began in the 2010s and maintained a steady growth trajectory. In the realm of histopathology, DL has found numerous applications spanning basic and advanced topics. Initially, the primary focus was on diagnosis, which then broadened to include prediction of phenotypic biomarkers such as cancer grade and subtypes. Over time, the scope extended to include molecular biomarkers, and ultimately evolved to encompass prognosis and drug response prediction. This diversification presents opportunities for more focused research on rare cancer entities, thereby enlarging our understanding of them. Conversely, the application of DL in genomics is currently less prevalent than in histopathology. The trend in genomics has leaned towards pan-cancer approaches with only a few publications investigating specific cancer types. However, there is the need to develop more cancer-type-specific diagnostic tests and prognostic biomarkers, thus paving the way for more personalized cancer care. Lastly, multimodal DL is a relatively new area that brings together data from both previously mentioned fields. Multimodal approaches have demonstrated the potential to outperform single-modality models, signifying its promising future. The synthesis of data from diverse sources, such as histopathology images and genomic sequences, offers a more comprehensive view of the disease, potentially leading to more accurate and clinically actionable insights. In conclusion, the dynamic evolution of DL in medical research, particularly in histopathology and genomics, underlines its potential in fostering breakthroughs in our understanding of diagnosis and treatment of cancer. Nevertheless, a considerable scope for further exploration and advancement remains. As the fields continue to grow and technology continues to improve, we expect that DL will play an increasing role in shaping the landscape of precision medicine.

### Supplementary Information

**Additional file 1. Supplementary Table 1. **Histopathology Papers. For all papers title, authors, year, journal and PubMed-URL, category, subcategory and cancer tissue are listed. **Supplementary Table 2. **Genomic Papers. For all papers title, authors, year, journal and PubMed-URL, category, subcategory and cancer tissue are listed. **Supplementary Table 3. **Multimodal Papers. For all papers title, authors, year, journal and PubMed-URL, category, subcategory and cancer tissue are listed. **Supplementary Table 4. **All Publications. For all papers title, authors, year, PubMed-URL and decision are listed. **Supplementary Table 5. **Timewise Publication Counts. Raw Counts of publications grouped by year and tissue type needed to generate Figure 2. **Supplementary Material. **Data selection of this study. Publications were collected from PubMed in nine search queries obtaining 3048 results. All papers were then uploaded to Rayyan to manually filter and classify them down to a total number of 534 articles used for this study.

## Data Availability

All data generated or analyzed during this study are included in this published article (and its supplementary information files).

## Abbreviations

AI: Artificial intelligence
DL: Deep learning
DNA: Deoxyribonucleic acid
HRD: Homologous repair deficiency
H&E: Hematoxylin and eosin
PARP: Poly-ADP-Ribose-Polymerase
PRISMA: Preferred Reporting Items for Systematic Reviews and Meta-Analyses
TCGA: The cancer genome atlas
WHO: World health organization
WSI: Whole slide image
