# Supplementary material for: A systematic analysis of deep learning in genomics and histopathology for precision oncology
Source: BMC Med Genomics. 2024 Feb 5;17:48. doi: 10.1186/s12920-024-01796-9 (PMC10845449; doi:10.1186/s12920-024-01796-9)
Supplement: Supplementary file 1 — Additional file 1. Supplementary Table 1. Histopathology Papers. For all papers title, authors, year, journal and PubMed-URL, category, subcategory and cancer tissue are listed. Supplementary Table 2. Genomic Papers. For all papers title, authors, year, journal and PubMed-URL, category, subcategory and cancer tissue are listed. Supplementary Table 3. Multimodal Papers. For all papers title, authors, year, journal and PubMed-URL, category, subcategory and cancer tissue are listed. Supplementary Table 4. All Publications. For all papers title, authors, year, PubMed-URL and decision are listed. Supplementary Table 5. Timewise Publication Counts. Raw Counts of publications grouped by year and tissue type needed to generate Figure 2. Supplementary Material. Data selection of this study. Publications were collected from PubMed in nine search queries obtaining 3048 results. All papers were then uploaded to Rayyan to manually filter and classify them down to a total number of 534 articles used for this study. [file 12920_2024_1796_MOESM1_ESM.zip › Supplementary Methods_ESM.docx]

# Supplementary Methods

### Search Terms

#### Histopathology

*Basic*

(deep learning histology[Title/Abstract]) OR (deep learning pathology[Title/Abstract]) OR (CNN pathology[Title/Abstract]) OR (CNN histology[Title/Abstract]) OR (RNN histology[Title/Abstract]) OR (RNN pathology[Title/Abstract]) OR (convolutional neural network histology[Title/Abstract]) OR (convolutional neural network pathology[Title/Abstract]) OR (recurrent neural network pathology[Title/Abstract]) OR (recurrent neural network histology[Title/Abstract]) OR (LSTM pathology[Title/Abstract]) OR (LSTM histology[Title/Abstract]) OR (GCN histology[Title/Abstract]) OR (graph convolutional network pathology[Title/Abstract]) OR (GCN pathology[Title/Abstract]) OR (graph convolutional network histology[Title/Abstract]) OR (GNN histology[Title/Abstract]) OR (graph neural network pathology[Title/Abstract]) OR (GNN pathology[Title/Abstract]) OR (graph neural network histology[Title/Abstract])

*Response*

(deep learning histology treatment response[Title/Abstract]) OR (deep learning pathology treatment response[Title/Abstract]) OR (CNN histology treatment response[Title/Abstract]) OR (CNN pathology treatment response[Title/Abstract]) OR (convolutional neural network histology treatment response[Title/Abstract]) OR (convolutional neural network pathology treatment response[Title/Abstract]) OR (recurrent neural network histology treatment response[Title/Abstract]) OR (recurrent neural network pathology treatment response[Title/Abstract]) OR (LSTM histology treatment response[Title/Abstract]) OR (LSTM pathology treatment response[Title/Abstract]) OR (RNN pathology treatment response[Title/Abstract]) OR (RNN histology treatment response[Title/Abstract]) OR (GCN histology treatment response[Title/Abstract]) OR (GCN pathology treatment response[Title/Abstract]) OR (graph convolutional network histology treatment response[Title/Abstract]) OR (graph convolutional network pathology treatment response[Title/Abstract]) OR (GNN histology treatment response[Title/Abstract]) OR (GNN pathology treatment response[Title/Abstract]) OR (graph neural network histology treatment response[Title/Abstract]) OR (graph neural network pathology treatment response[Title/Abstract])

*Survival*

(deep learning histology survival[Title/Abstract]) OR (deep learning pathology survival[Title/Abstract]) OR (CNN histology survival[Title/Abstract]) OR (CNN pathology survival[Title/Abstract]) OR (convolutional neural network histology survival[Title/Abstract]) OR (convolutional neural network pathology survival[Title/Abstract]) OR (recurrent neural network histology survival[Title/Abstract]) OR (recurrent neural network pathology survival[Title/Abstract]) OR (LSTM histology survival[Title/Abstract]) OR (LSTM pathology survival[Title/Abstract]) OR (RNN histology survival[Title/Abstract]) OR (RNN pathology survival[Title/Abstract]) OR (GCN histology survival[Title/Abstract]) OR (GCN pathology survival[Title/Abstract]) OR (graph convolutional network histology survival[Title/Abstract]) OR (graph convolutional network pathology survival[Title/Abstract]) OR (GNN histology survival[Title/Abstract]) OR (GNN pathology survival[Title/Abstract]) OR (graph neural network histology survival[Title/Abstract]) OR (graph neural network pathology survival[Title/Abstract])

*Mutation*

(deep learning histology mutation[Title/Abstract]) OR (deep learning pathology mutation[Title/Abstract]) OR (CNN histology mutation[Title/Abstract]) OR (CNN pathology mutation[Title/Abstract]) OR (convolutional neural network histology mutation[Title/Abstract]) OR (convolutional neural network pathology mutation[Title/Abstract]) OR (recurrent neural network histology mutation[Title/Abstract]) OR (recurrent neural network pathology mutation[Title/Abstract]) OR (RNN histology mutation[Title/Abstract]) OR (RNN pathology mutation[Title/Abstract]) OR (LSTM histology mutation[Title/Abstract]) OR (LSTM pathology mutation[Title/Abstract]) OR (GCN histology mutation[Title/Abstract]) OR (GCN pathology mutation[Title/Abstract]) OR (graph convolutional network histology mutation[Title/Abstract]) OR (graph convolutional network pathology mutation[Title/Abstract]) OR (GNN histology mutation[Title/Abstract]) OR (GNN pathology mutation[Title/Abstract]) OR (graph neural network histology mutation[Title/Abstract]) OR (graph neural network pathology mutation[Title/Abstract])

#### Genomics

*Basic*

(deep learning genomics[Title/Abstract]) OR (CNN genomics[Title/Abstract]) OR (convolutional neural network genomics[Title/Abstract]) OR (RNN genomics[Title/Abstract]) OR (recurrent neural network genomics[Title/Abstract]) OR (LSTM genomics[Title/Abstract]) OR (GCN genomics[Title/Abstract]) OR (graph convolutional network genomics[Title/Abstract]) OR (GNN genomics[Title/Abstract]) OR (graph neural network genomics[Title/Abstract])

*Response*

(deep learning genomics treatment response[Title/Abstract]) OR (CNN genomics treatment response[Title/Abstract]) OR (convolutional neural network genomics treatment response[Title/Abstract]) OR (recurrent neural network genomics treatment response[Title/Abstract]) OR (LSTM histology treatment response[Title/Abstract]) OR (RNN genomics treatment response[Title/Abstract]) OR (GCN genomics treatment response[Title/Abstract]) OR (graph convolutional network genomics treatment response[Title/Abstract]) OR (GNN genomics treatment response[Title/Abstract]) OR (graph neural network genomics treatment response[Title/Abstract])

*Survival*

(deep learning genomics survival[Title/Abstract]) OR (CNN genomics survival[Title/Abstract]) OR (convolutional neural network genomics survival[Title/Abstract]) OR (recurrent neural network genomics survival[Title/Abstract]) OR (LSTM histology survival[Title/Abstract]) OR (RNN genomics survival[Title/Abstract]) OR (GCN genomics survival[Title/Abstract]) OR (graph convolutional network genomics survival[Title/Abstract]) OR (GNN genomics survival[Title/Abstract]) OR (graph neural network genomics survival[Title/Abstract])

*Mutation*

(deep learning genomics mutation[Title/Abstract]) OR (CNN genomics mutation[Title/Abstract]) OR (convolutional neural network genomics mutation[Title/Abstract]) OR (recurrent neural network genomics mutation[Title/Abstract]) OR (LSTM histology mutation[Title/Abstract]) OR (RNN genomics mutation[Title/Abstract]) OR (GCN genomics mutation[Title/Abstract]) OR (graph convolutional network genomics mutation[Title/Abstract]) OR (GNN genomics mutation[Title/Abstract]) OR (graph neural network genomics mutation[Title/Abstract])

#### Multimodality

(multi-modal pathology genomics deep learning[Title/Abstract]) OR (multi-modal pathology genomics deep learning[Title/Abstract]) OR (multi-modal histology genomics deep learning[Title/Abstract]) OR (multi-modal histology genomics deep learning[Title/Abstract]) OR (multimodal pathology genomics deep learning[Title/Abstract]) OR (multimodal pathology genomics deep learning[Title/Abstract]) OR (multimodal histology genomics deep learning[Title/Abstract]) OR (multimodal histology genomics deep learning[Title/Abstract]) OR (multi-modal pathology genomics DL[Title/Abstract]) OR (multi-modal pathology genomics DL[Title/Abstract]) OR (multi-modal histology genomics DL[Title/Abstract]) OR (multi-modal histology genomics DL[Title/Abstract]) OR (multimodal pathology genomic DL[Title/Abstract]) OR (multimodal pathology genomics DL[Title/Abstract]) OR (multimodal histology genomics DL[Title/Abstract]) OR (multimodal histology genomics DL[Title/Abstract])
